# Impact of genetic polymorphisms of drug transporters *ABCB1* and *ABCG2* and regulators of xenobiotic transport and metabolism *PXR* and *CAR* on clinical efficacy of dasatinib in chronic myeloid leukemia

**DOI:** 10.3389/fonc.2022.952640

**Published:** 2022-09-23

**Authors:** Anna Marta Madejczyk, Federico Canzian, Joanna Góra-Tybor, Daniele Campa, Tomasz Sacha, Dorota Link-Lenczowska, Izabela Florek, Witold Prejzner, M. Całbecka, M. Rymko, M. Dudziński, Magdalena Julita Orzechowska, Krzysztof Jamroziak

**Affiliations:** ^1^ Department of Hematology, Medical University of Łódź, Łódź, Poland; ^2^ Genomic Epidemiology Group, German Cancer Research Center Deutsche Krebsforschungszentrum (DKFZ), Heidelberg, Germany; ^3^ Department of Biology, University of Pisa, Pisa, Italy; ^4^ Department of Hematology, Jagiellonian University Medical College, Kraków, Poland; ^5^ Department of Hematology, Medical University of Gdańsk, Gdańsk, Poland; ^6^ Department of Hematology, Copernicus Specialist Municipal Hospital, Toruń, Poland; ^7^ Department of Hematology, Teaching Hospital No 1, Rzeszów, Poland; ^8^ Department of Molecular Carcinogenesis, Chair of Molecular Medicine and Biotechnology, Faculty of Medicine, Medical University of Łódź, Łódź, Poland; ^9^ Department of Hematology, Transplantation and Internal Medicine, Medical University of Warsaw, Warsaw, Poland

**Keywords:** Chronic myeloid leukemia, dasatinib, ABCB1, ABCG2, PXR, CAR, single nucleotide polymorphisms

## Abstract

**Introduction:**

Functional single-nucleotide polymorphisms (SNPs) in genes regulating cellular uptake, elimination, and metabolism of xenobiotics may potentially influence the outcome of chronic myeloid leukemia (CML) patients treated with BCR-ABL1 tyrosine kinase inhibitors (TKI). Dasatinib, a second-generation TKI, is a substrate of the ABC-superfamily xenobiotic transporters ABCB1 (MDR1, Pg-P) and ABCG2 (BCRP). Pregnane X receptor (PXR, NR1I2) and constitutive androstane receptor (CAR, NR1I3) are involved in the control of expression of *ABCB1* and *ABCG2.*

**Aim of the study:**

In this study, we assessed the impact of inherited variants in *ABCB1*, *ABCG2*, *PXR*, and *CAR* genes on dasatinib efficacy and toxicity in CML.

**Materials and methods:**

Sixty-one tagging SNPs in *ABCB1*, *ABCG2*, *PXR*, and *CAR* genes were analyzed by real-time quantitative PCR with specific probes in 86 CML patients who failed imatinib therapy.

**Results:**

We found the associations between SNPs rs7787082 (*ABCB1*, OR = 0.2; 95% CI = 0.06-0.66, p = 0.008), rs12505410 (*ABCG2*, OR = 3.82; 95% CI = 1.38-10.55; p = 0.010), and rs3114018 (*ABCG2*, OR = 0.24; 95% CI = 0.08-0.71; p = 0.010) and the probability of achieving CCyR. Furthermore, progression-free survival (PFS) was significantly influenced by SNPs rs3732357 (HR = 0.2, 95% CI = 0.26-0.70; p = 0.001), rs3732360 (HR = 0.59; 95% CI = 0.38-0.93; p = 0.020), rs11917714 (HR = 0.58; 95% CI = 0.36-0.92; p = 0.020), and rs3732359 (HR = 0.57; 95% CI = 0.36-0.91; p = 0.024) in *PXR*; rs2307418 (HR = 2.02; 95% CI = 1.19-3.43; p = 0.048) in *CAR*; and rs2235023 (HR = 2.49; 95% CI = 1.13-5.50; p = 0.011) and rs22114102 (HR = 1.90; 95% CI = 1.00-3.63; p = 0.028) in *ABCB1*. Moreover, overall survival (OS) was impacted by rs3842 (HR = 1.84; 95% CI = 1.01-3.33; p = 0.012) and rs2235023 (HR = 2.28; 95% CI = 1.03 = 5.02; p = 0.027) in *ABCB1*, rs11265571 (HR = 1.59; 95% CI = 0.82-3.08; p = 0.037) and rs2307418 (HR = 73.68; 95% CI = 4.47-1215.31; p = 0.003) in *CAR*, and rs3732360 (HR = 0.64; 95% CI = 0.40 = 1.04; p = 0.049) in *PXR*. Taking into account the influence of the tested SNPs on treatment toxicity, we found a significant relationship between allele G of polymorphism in the *ABCB1* rs7787082 (OR = 4.46; 95% CI = 1.38-14.39 p = 0.012) and hematological complications assuming the codominant gene inheritance model as well as a significant correlation between the presence of minor allele (G) of SNP rs2725256 in the *ABCG2* gene (OR = 4.71; 95% CI = 1.20-18.47; p = 0.026) and the occurrence of non-hematological complications assuming a recessive gene inheritance model.

**Conclusion:**

Our data suggest that inherited variants in the genes encoding for proteins involved in the transport of xenobiotics may modify the toxicity and efficacy of dasatinib therapy in CML patients.

## Introduction

Chronic myelogenous leukemia (CML) is a rare hematological malignancy with an yearly incidence of one per 100,000 individuals (1). The molecular background of the disease has been elucidated since the discovery of the Philadelphia (Ph) chromosome, a consequence of the reciprocal translocation between chromosomes 9 and 22 and the resulting *BCR-ABL1* fusion gene that encodes for constitutively activated tyrosine kinase (2, 3). Introduction of imatinib, the first-generation BCR-ABL1 tyrosine kinase inhibitor (TKI), has been a great proof of concept of targeted therapy and has dramatically improved CML prognosis (4). However, approximately one-third of patients develop primary or secondary resistance to imatinib (5). Second-generation TKIs such as dasatinib, and later nilotinib and bosutinib, were introduced to treat imatinib-resistant or intolerant CML patients and subsequently also become first-line treatment options (6, 7).

Dasatinib has a different chemical structure than imatinib and higher potential to inhibit BCR-ABL1 kinase (8). The drug targets a broader spectrum of kinases including SRC kinases, and this contributes to its efficacy as well as specific side effects in CML (9). The pharmacokinetic properties of dasatinib are similar to other TKIs. After oral administration, the molecule binds plasma proteins and is metabolized by liver P450 cytochrome family enzymes (mainly CYP3A4) as well as FMO-3 and UDT-glucuronylotransferase. The process of dasatinib excretion from cells is mediated by ATP-binding cassette (ABC) transporters ABCB1 and ABCG2 (10). ABC transporters are a conserved family of membrane proteins responsible for cell protection against xenobiotics (11).

The expression of genes involved in metabolism and excretion of xenobiotics including the ABC genes is regulated by specific xenosensors—genes that are activated as a response to higher concentrations of xenobiotics. Among them, *PXR* (*NR1I2*) and *CAR* (*NR1I3*) possess specific DNA-binding domains built up from zinc fingers which enable them to recognize DNA elements, characteristic for enzymes taking part in metabolism of xenobiotic substances (12, 13). PXR and CAR stimulate the expression not only of ABCB1 and ABCG2 but also of cytochrome P450 enzymes and many other genes (14, 15). It was shown that single-nucleotide polymorphisms (SNPs) in the aforementioned genes impact on the metabolism of various drugs (16, 17).

The present study aimed to define genetic markers influencing the outcome of dasatinib therapy in patients with imatinib-resistant or intolerant CML. To that end, we analyzed 61 tagging SNPs in *ABCB1, ABCG2, PXR*, and *CAR* genes and studied their effects regarding different parameters of response depth and duration as well as toxicity in CML patients.

## Materials and methods

### Patients

The study included 86 Polish Caucasian CML patients treated in five tertiary hematological centers in Poland (Department of Hematology, Medical University of Łódź; Department of Hematology, Jagiellonian University Medical College, Kraków; Department of Hematology, Medical University of Gdańsk; Department of Hematology, Copernicus Specialist Municipal Hospital, Toruń; Department of Hematology, Teaching Hospital No 1, Rzeszów). The group included 43 women and 43 men with a median age of 48 years at CML diagnosis (range 18-100). All patients received imatinib at 400 mg/day as a first-line treatment with TKI. The initial dose of dasatinib administered in the second (after imatinib failure) or third (after imatinib and nilotinib failure) line of therapy was 100 mg/day. Complete clinical and laboratory data concerning the course of dasatinib therapy were collected for the analyses of the association with the tested SNPs. Cytogenetic responses were evaluated by classical chromosome banding technique or FISH. The *BCR-ABL1* gene expression was assessed by quantitative real-time PCR according to standard protocols described elsewhere (18).

### Clinical endpoints

For the purpose of this analysis, we used definitions of treatment endpoints consistent with the European Leukemia Net recommendations (19). Cytogenetic response (CyR) was defined as complete (0% of Ph chromosome, CCyR), partial (1%–34% of Ph chromosome, PCyR), minimal (35%–65% Ph chromosome, mCyR), and no response (>65% of Ph chromosome). Optimal cytogenetic response was categorized as CCyR achievement within 12 months from treatment start. Molecular responses were defined as complete (CMR) when the *BCR-ABL1* gene transcript level was below 0.01%, major (MMR) when the *BCR-ABL1* gene transcript level was between 0.01% and 0.1%, and no response when the *BCR-ABL1* gene transcript level was >0.1%. Optimal molecular response was defined as achievement of at least MMR within 18 months from treatment start. Progression-free survival (PFS) was defined as the interval between start of dasatinib treatment and CML progression or death from any cause, or last follow-up without progression. Overall survival (OS) was counted as the time between dasatinib treatment start and date of death, or last follow-up when the patient was still alive. Analyzed adverse events of the treatment with dasatinib included hematological toxicities of grade 3 or 4 and any non-hematological complication of grades 2–4.

### Polymorphism selection

Tagging SNPs for *ABCB1*, *ABCG2*, *PXR*, and *CAR* were selected using the Tagger algorithm, available through Haploview (20), using pairwise SNP selection with a minimum r^2^ threshold of 0.8.

The set of common genetic variants (sequences including 5 kb upstream of the first exon and 5 kb downstream of the last exon of each gene), with minor allele frequency (MAF) ≥5% in Caucasians from the International HapMap Project (21), was included for *ABCB1*, *ABCG2*, *PXR*, and *CAR.* This process resulted in a selection of 26 tagging SNPs for *ABCB1* (average r^2^ of tagging SNPs with the SNPs they tag = 0.958), 17 SNPs for *ABCG2* (average r^2^ = 0.965), 11 SNPs for *PXR* (average r^2^ = 0.975), and seven SNPs for *CAR* (average r^2^ = 1.000). This selection therefore captures a very high degree of the known common variability in these genes (22).

### Genotyping

DNA was isolated from peripheral blood samples using DNA Blood Mini Kit (Qiagen) and genotyped. Duplicates of 8% of the samples were interspersed throughout the plate for ensuring the internal quality controls. Both TaqMan (ABI, Applied Biosystems, Foster City, CA, USA) and KASP (KBioscence, Hoddesdon, UK) technologies were used for genotyping according to the manufacturer’s protocol. PCR plates for TaqMan as well as KASP assays were read on a Viia7 Real-Time PCR platform (Applied Biosystems). The Viia7 RUO Software (Applied Biosystems) was used to determine the genotypes. In our analysis, all individuals with a call rate <80% were excluded from further investigation.

### Statistical analysis

Statistical significance of the associations between selected parameters of response to dasatinib therapy and genetic variants was evaluated using R v2.11 software. Logistic regression was used to assess the association between the genetic variability of the SNPs and treatment efficacy, defined by the following endpoints: CCyR at 12 months, MMR at 18 months. Treatment toxicity was investigated with logistic regression as well, using the following endpoints: appearance of any non-hematological toxicities of grades 2–4 including fluid retention, hematological toxicity of grade 3 or 4. These endpoints were used as dichotomous variables. To study the associations between SNPs and PFS and OS, Cox proportional hazard regression was used. SNPs were analyzed according to the following inheritance models: “co-dominant,” where the homozygous major allele (reference category) was compared separately with two different genotypes that include the minor allele (heterozygotes and homozygotes for the variant allele), and “recessive,” whereby the comparison groups were minor homozygous genotypes against the rest (combining heterozygotes and homozygotes for the major allele). The most significant test between the codominant and the recessive genetic models was used to determine the statistical significance of each association of each SNP. All analyses were adjusted by age at diagnosis, sex, CML phase, Sokal score, and use of dasatinib in the second or third line. The results of logistic regression analyses were expressed as odds ratios (OR), and the results of Cox regression analyses were expressed as hazard ratios (HR), with 95% confidence intervals (CI). The results were adjusted for multiple comparisons by Bonferroni correction which was calculated as p = 0.05/(61 SNPs × 2 models) = 0.00041.

### Functional characterization of the SNPs

For the SNPs that achieved statistical significance, several online databases were used for analysis of the influence of SNPs on cellular processes such as transcription or association with protein activity.

RegulomeDB is a database that assigns SNPs to predicted and known regulatory sites in the human genome regions that affect transcriptional processes, DNAase hypersensitivity regions, transcription factor binding sites, and promoter regions.

HaploReg is a tool designed to search for significant effects of SNPs in haplotype blocks, including SNPs in disease-related loci, on gene expression levels and protein activity. Using HaploReg, it is possible to assess whether specific SNPs are located in the eQTL (expression quantitative trait loci) loci and to check the effect of SNPs on gene regulatory regions. GTEx is a database that allows to search for relationships between genetic variation (including SNPs) and the expression of genes and proteins in individual tissues.

## Results

### Clinical data

Eighty-six Polish Caucasian CML patients treated in five tertiary hematological centers in Poland were genotyped within the present study. The characteristics of the study population are described in **Table 1**. The majority of patients was diagnosed in the chronic phase (85%). Only 6% of the patients carried additional cytogenetic aberrations; the remainder were characterized only by the presence of the Ph chromosome. Regarding Sokal score, 43% of patients were diagnosed as low risk, and 31% and 26% were characterized as intermediate and high risk, respectively.

**Table 1:** Summary of the dasatinib treatment results.

Dasatinib treatment	*Number of patients (%)*
**All**	86 (100%)
In the 2nd line	78 (90.7%)
In the 3rd line	8 (9.3%)
Response to treatment^a^	
Optimal CyR^b^	52 (60.5%)
CCyR	49 (57%)
PCyR	9 (11%)
mCyR	21 (24%)
noCyR	7 (8%)
Optimal MR^c^	8 (9.3%)
CMR	24 (30%)
MMR	19 (24%)
No MR	37(46%)
**Progression**	14 (16.3%)
**Fluid retention**	15 (17.4%)
**Incidence** **of any hematological complications**	33 (38.4%)

a Response to treatment: in the database were included results of the last medical visit for each patient.

b Optimal CyR: achievement of the complete cytogenetic response within 12 months from treatment start.

c Optimal MR: achievement of at least the major molecular response within 18 months from treatment start. Only eight patients (9.3%) achieved optimal response, i.e., at least MR3 within 18 months from the start of treatment; other patients achieved CMR and MMR but after 18 months of treatment, therefore they were not included as optimal MR.

Data on the cytogenetic and molecular response as well as the occurrence of treatment side effects are included.

**Figure 1:**
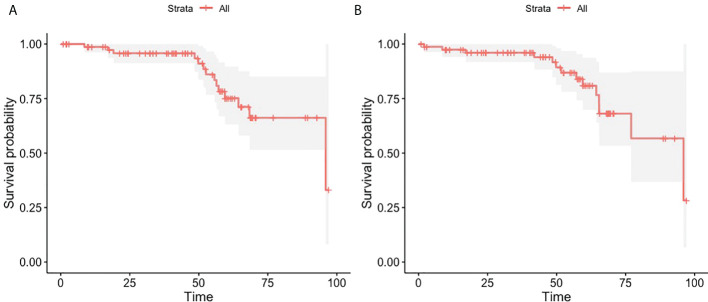
The Kaplan-Meier plots showing overall survival **(A)** and progression- free survival **(B)** in the whole cohort of 86 CML patients treated with dasatinib.

Eighteen patients (21%) discontinued imatinib due to intolerance to the treatment, 68 patients (79%) due to disease progression. The median duration of imatinib treatment in these patients was 637 days (1.75 years), while the median duration of dasatinib treatment, calculated as the time between the initiation of therapy and the last medical check-up, was 1,492 days (4.09 years). The overall survival and PFS of the cohort is showed in **Figure 1**.

All patients received imatinib as first-line therapy of CML in the standard dose of 400 mg once daily and were later treated with dasatinib at 100 mg/day due to the ineffectiveness or intolerance of imatinib. Patients treated with dasatinib right after imatinib treatment (in the second line) accounted for 91% (78 patients) of the group, while eight patients (9%) received dasatinib in the third line, after nilotinib was given for imatinib failure or toxicity.

Sixty-five patients (76%) had no changes in dasatinib dosage while in five cases (6%) the dose was subsequently increased to 140 mg/day at the physician’s discretion and 16 (18%) patients had the dose decreased to 80 mg/day due to toxicity. The main complications that occurred during the follow-up in the present analysis were fluid retention (15 patients, 17%) and hematological complications including thrombocytopenia, cytopenia, neutropenia, lymphopenia, and agranulocytosis (33 patients, 38%). Other undesirable effects included pulmonary hypertension, abdominal pain, arrhythmias, and increased creatine kinase level.

### Genotyping quality control

Genotyping of 61 preselected SNPs for *ABCB1*, *ABCG2*, *CAR*, and *PXR* was successful in all included patients. The average call rate was 98.42% ranging from 85.25% to 100%. Genotype distributions were in accordance with the Hardy–Weinberg equilibrium for all tested loci.

### Influence of SNPs on cytogenetic and molecular response to dasatinib

The analysis using a logistic regression model identified the significant impact of tested SNPs on the probability of achieving CCyR following dasatinib treatment. Assuming the codominant model of inheritance, noteworthy associations (p < 0.05) were found between the achievement of CCyR after 12 months of therapy and the following SNPs: *ABCB1* gene: rs7787082, *PXR* rs2461818 and two SNPs in the *ABCG2* gene: rs12505410 and rs3109823 (**Table 2**). Assuming a recessive inheritance model, statistical significance was observed for two SNPs in the *ABCG2* gene: rs2622621 and rs3114018 (**Table 2**). No significant results were found regarding the potential influence of SNPs on the probability of the achievement of MMR after 18 months of dasatinib treatment.

**Table 2:** Associations between selected SNPs and cytogenetic response after 12 months of treatment as well as incidence of hematological and non-hematological complications.

SNP	Gene	Alleles M/m^a^	Endpoint^b^	Model^c^	OR	95% CI	p-value
rs7787082	*ABCB1*	G/A	CyR-12	CD	0.20	0.06-0.66	0.008
rs2461818	*PXR*	C/T	CyR-12	CD	0.16	0.03-0.89	0.036
rs12505410	*ABCG2*	T/G	CyR-12	CD	3.82	1.38-10.55	0.010
rs3109823	*ABCG2*	T/C	CyR-12	CD	2.87	1.11-7.40	0.029
rs2622621	*ABCG2*	C/G	CyR-12	R	0.21	0.05-0.92	0.038
rs3114018	*ABCG2*	A/C	CyR-12	R	0.24	0.08-0.71	0.010
rs7787082	*ABCB1*	G/A	HC	CD	4.46	1.38-14.39	0.012
rs2725256	*ABCG2*	A/G	NHC	R	4.71	1.20-18.47	0.026

a M, major allele; m, minor allele.

b CyR-12, achievement of cytogenetic response within 12 months from treatment start; HC, hematological complications; NHC, non-hematological complications.

c CD, codominant; R, recessive.

### Influence of SNPs on dasatinib therapy side effects

There was no significant association between tested clinical factors and the occurrence of analyzed side effects including grade 2–4 non-hematological complications or grade 3 and 4 neutropenia or thrombocytopenia. In contrast, a noteworthy correlation was observed between rs7787082, in the *ABCB1*, and the occurrence of grade 3 or 4 hematological complications assuming the codominant inheritance model. Furthermore, a significant association was found between rs2725256 in *ABCG2* and occurrence of non-hematological complications assuming a recessive model of inheritance (**Table 2**).

### Influence of SNPs on OS and PFS on dasatinib therapy

We found a statistically significant influence of Sokal score on OS (HR = 1.34, 95% CI = 1.21-2.09, p = 0.001). No other clinical pretreatment parameter was related to patients’ survival functions. Interestingly, assuming the codominant inheritance model, there was a significant correlation between four SNPs (*ABCB1* rs3842, *ABCB1* rs2235023, *CAR* rs11265571, *PXR* rs3732360) and OS in the patients treated with dasatinib. Furthermore, rs2307418 in the *CAR* gene impacted OS assuming a recessive inheritance model.

**Figure 2:**
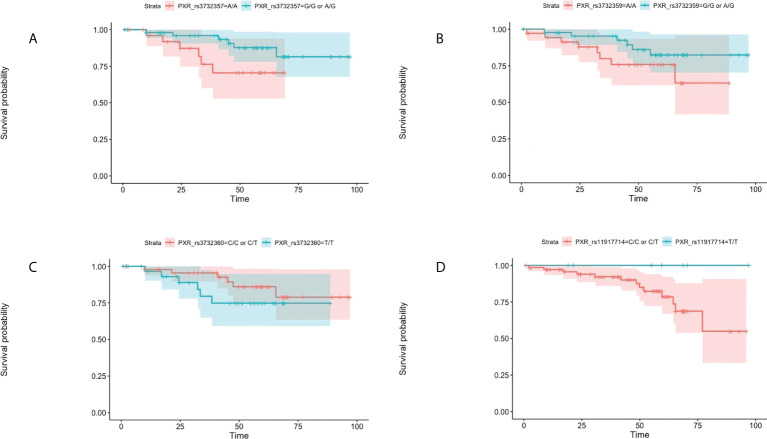
Kaplan-Meier plots for progression- free survival in regard to the presence of rs3732357 **(A)**, rs3732359 **(B)**, rs3732360 **(C)**, and rs11917714 **(D)** in the PXR gene in the co-dominant inheritance model.

Moreover, seven tested SNPs significantly influenced the probability of PFS. Assuming the codominant model of inheritance, these were the following SNPs: *CAR* rs2307418 *PXR* rs3732357, *ABCB1* rs2235023 *ABCB1* rs22114102 *PXR* rs3732360, and *PXR* rs11917714 *PXR* rs3732359 (**Table 3** and **Figure 2**).

**Table 3:** Associations between selected SNPs, overall survival, and progression-free survival during dasatinib treatment.

SNP	Gene	Alleles M/m^a^	Endpoint^b^	Model^c^	HR	95%CI	p value
rs3842	*ABCB1*	T/C	OS	CD	1.84	1.01-3.33	0.012
rs2235023	*ABCB1*	C/T	OS	CD	2.28	1.03-5.02	0.027
rs11265571	*CAR*	A/T	OS	CD	1.59	0.82-3.08	0.037
rs3732360	*PXR*	T/C	OS	CD	0.64	0.40-1.04	0.049
rs2307418	*CAR*	T/G	OS	R	73.68	4.47-1215.31	0.003
rs2307418	*CAR*	T/G	PFS	CD	2.02	1.19-3.43	0.048
rs3732357	*PXR*	A/G	PFS	CD	0.42	0.26-0.70	0.001
rs2235023	*ABCB1*	C/T	PFS	CD	2.49	1.13-5.50	0.011
rs22114102	*ABCB1*	C/T	PFS	CD	1.90	1.00-3.63	0.028
rs3732360	*PXR*	T/C	PFS	CD	0.59	0.38-0.93	0.020
rs11917714	*PXR*	C/T	PFS	CD	0.58	0.36-0.92	0.020
rs3732359	*PXR*	A/G	PFS	CD	0.57	0.36-0.91	0.024

a M, major allele; m, minor allele.

b OS, overall survival; PFS, progression-free survival.

c CD, codominant; R, recessive.

### Functional SNP annotation

**Table 4** shows a summary of the potential functional impact of the tested SNPs based on HaploReg, RegulomeDB, and GTEx databases. The table includes SNPs that showed statistical importance in this analysis.

**Table 4:** Summary of bioinformatic SNP annotations.

SNP ID	Gene	Alleles M/m^a^	MAF^b^	Rank^c^	HaploReg^d^	eQTL^e^	GTEx^f^
rs7787082	*ABCB1*	G/A	0.18	6	*CRX FOXD3 GFI1*	–	Testes, skin
rs3842	*ABCB1*	T/C	0.14	n/a	*PLZF*	*ABCB4*	Brain, nerves
rs2235023	*ABCB1*	C/T	0.09	6	*CEBPB ISL2 POU1F1 RHOX11*	*ABCB1 ABCB4*	Testes, muscles
rs2214102	*ABCB1*	C/T	0.09	4	*GR*	–	Heart, colon
rs2725256	*ABCG2*	A/G	0.33	6	*FOXA HNF4 HBP1* *POU1F1 RXRA STAT*	*SPP1*	Adipocytes
rs12505410	*ABCG2*	T/G	0.40	4	*PBX3*	–	Testes, blood
rs2622621	*ABCG2*	C/G	0.30	5	*-*	–	Blood
rs3114018	*ABCG2*	A/C	0.49	5	*LHX3 MEF2 NANOG POU2F2*	–	Esophagus, heart
rs3109823	*ABCG2*	T/C	0.26	6	*CTCF NRF-2 YY1*	–	Blood
rs2307418	*CAR*	T/G	0.15	5	*ERalpha-A GCMGR*	*TOMM40L USF1*	Brain, muscles, skin
rs11265571	*CAR*	A/T	0.17	4	*ERalpha-A PAX-5 RHOX11*		Skin, testes, colon
rs2461818	*PXR*	C/T	0.08	6	*ARID5B FOXO2 FOXO3 FOXP1 HDAC2 IK2 PLZF SOX6*	*PLA1A GSK3B*	Thyroid
rs11917714	*PXR*	C/T	0.17	6	*HIC1*	*GSK3B*	Nerves, small intestine, esophagus, testes
rs3732357	*PXR*	A/G	0.35	5	*GLI GLIS2 ZIC*	GSK3B	Adipocytes, colon, esophagus, stomach, arteries, thyroid, brain, muscles, lungs
rs3732360	*PXR*	T/C	0.23	6	*E2A LMO2 MYF TATA*	*GSK3B GPR156*	Adipocytes, nerves, esophagus, brain, colon, arteries
rs3732359	*PXR*	A/G	0.20	6	*BCL NRSF PLAG1* *SIN3AK20 TAL1 YY1*	*GSK3B GPR156*	Adipocytes, nerves, esophagus, brain, colon, arteries, thyroid

The table contains associations for all SNPs, while those that occur only in blood cells are bold.

a M: major allele; m: minor allele.

b MAF: minor allele frequency in the 1000 Genomes European population.

c Rank from RegulomeDB: 1 is given to SNPs showing the strongest evidence of a role in regulating the transcription process by binding transcription factors, while 6 to SNPs with a low probability of influencing to transcription.

d HaploReg: the tested SNP probably influences the expression of mentioned genes.

e eQTL: the tested SNP is located in the eQTL (expression quantitative trait loci) of mentioned genes.

f GTEx: the relationship between the tested SNP and the tissue in which the gene is expressed.

The RegulomeDB portal achieving scores 1–3 indicates the probability of the analyzed SNP belonging to the sequence affecting the binding process of transcription factors. All tested SNPs scored 4, 5, and 6, which means that the binding of transcription factors is very unlikely.

Analysis with HaploReg showed the probable influence of SNPs on the regulation of genes. Noteworthy is the influence of some studied SNPs on the family of FOX transcription factors, which affect a number of cellular processes. All details are included in **Table 4**.

In the present work using the GTEx portal, a significant link with the gene expression level was demonstrated for most of the candidate SNPs tested. However, only for SNPs rs12505410, rs2622621, rs3109823 in *ABCG2* were the associations in blood cells, while the rest were in tissues not related to CML pathogenesis.

## Discussion

We used a candidate gene approach to evaluate the impact of inherited genetic differences on the outcome of CML treatment with dasatinib. The *ABCB1* and *ABCG2* genes, encoding for known transporters of dasatinib, and *PXR* and *CAR* xenosensor genes, with the role of transcription factors for many genes which take part in the pharmacokinetic processes, were chosen for analysis.

A tagging approach was applied to capture the common genetic variability of *ABCB1*, *ABCG2*, *PXR*, and *CAR* and resulted in a selection of a total of 61 tagging SNPs that were subsequently genotyped in 86 CML patients treated with dasatinib in the second and third lines of treatment.

Significant amount of data has been reported regarding the influence of SNPs in drug transport and metabolism genes on imatinib, another TKI. Kim et al. investigated the influence of SNPs in genes potentially involved in metabolism of imatinib. They found that the rs2231137 GG homozygotes (*ABCG2*), rs776746 AA (*CYP3A5*) homozygotes, and advanced stage strongly correlated with poor response to treatment with imatinib; however, the *SLC22A1*-rs683369 GG homozygotes and advanced stage were correlated with therapy failure (23). Seong et al. analyzed the impact of SNPs in cytochrome P450 enzymes and drug transporters on the imatinib concentration in plasma and clinical response in CML patients. They concluded that rs2231142 (421C>A) situated in *ABCG2* is highly associated with MMR achieved by CML patients (24). In contrast, Takahashi et al. showed that homozygotes AA in the same SNP had a higher imatinib concentration than CC (25). In our study, rs2231142 (421C>A) in *ABCG2* showed no statistically significant association on dasatinib treatment endpoints.

However, to the best of our knowledge there are little data available regarding influence on inherited background in ABC transporters on dasatinib therapy in CML. Skoglund et al. found that wild-type ABCG2 had a protective effect against the cytotoxicity of all investigated tyrosine kinase inhibitors in exception of bosutinib. Skoglund et al.’s finding of SNPs *ABCG2* 421C>A, 623T>C, 886G>C, and 1574T>G showed a reduction in *ABCG2* cell membrane expression and the protective effect of *ABCG2* against imatinib, CGP74588, dasatinib, and nilotinib cytotoxicity (26). In our study, rs2231142 (421C>A) showed no significance regarding dasatinib treatment endpoints.

To our knowledge, there are no publications available on the effect of single-nucleotide polymorphisms in the *PXR* and *CAR* nuclear receptor genes on the outcomes of dasatinib treatment in CML patients. However, based on the results of our work, it is assumed that such an influence exists. Analysis of SNPs showed that rs2461818 in *PXR* has an influence on achieving clinical outcome expressed through CCyR after 12 months (OR = 0.16; 95% CI = 0.03-0.89; p = 0.036). The CAR and PXR proteins belonging to the same family of nuclear receptors are known as transcription factors for genes involved in the metabolism of exogenous substances and their removal from the body (13). As CAR stimulates the expression of proteins related to imatinib metabolism ABCB1, ABCG2, ABCC2, hOCT1, and CYP3A4 (13, 27, 28), genetically influenced changes in the activity of this gene product may have an indirect effect on the plasma concentration of imatinib and thus potentially also the effects of TKI BCR-ABL1 treatment. Although dasatinib belongs to the same group of drugs, its molecular structure and metabolism differ from that of imatinib, which may result in a different response to treatment in the context of the same polymorphic changes, as evidenced by the obtained results.

Loscocco et al., in their study, examined SNPs in genes from the ABC family (*ABCB1*, *ABCG2*, *ABCC1*, *ABCC2*) to determine their effectiveness in treatment with another tyrosine kinase inhibitor—nilotinib—in a group of 90 CML patients. They found that CC and CT genotypes in *ABCC2* rs3740066 as well as the TT genotypes in *ABCB1* rs1045642 correlated with a higher probability of achieving MR3 in a shorter time (*p* = 0.02, *p* = 0.004, and *p* = 0.01), where the GG genotype of *ABCG2* rs2231137 was associated with a lower probability of MR3 achievement (*p* = 0.005). Moreover, the *ABCC2* rs3740066 CC genotype and the *ABCB1* rs1045642 CC and TT genotypes were positively correlated with MR4 achievement (*p* = 0.02, *p* = 0.007, and *p* = 0.003) (29). Our study does not reveal any correlations between examined SNPs and molecular response in patients treated with dasatinib.

Our results suggest that the naturally occurring germline variation in tested genes has influence on such important endpoints of dasatinib therapy in CML as well as probability of cytogenetic and molecular responses, PFS and OS.

To the best of our knowledge, this is the first study on the impact of *ABCB1*, *ABCG2*, *PXR*, and *CAR* gene polymorphisms on the outcomes of dasatinib treatment in CML. The work clearly shows the influence of SNPs present in genes related to dasatinib metabolism (*ABCB1* and *ABCG2*) and genes encoding transcription factors (*PXR*, *CAR*) on treatment outcomes, susceptibility to side effects, and overall survival and progression-free time. Furthermore, the analysis we present here has a number of strengths. First of all, cases were collected in a relatively small number of hematological centers with high medical reference. Information about multiple clinical endpoint variables of patients has been thoroughly compiled in a single database. For each patient, detailed clinical history and treatment history were checked. The collected data showed that the whole group was homogenous, especially in terms of lack of a good response to imatinib. To the best of our knowledge, this is the first study on the impact of *ABCB1*, *ABCG2*, *PXR*, and *CAR* gene polymorphisms on the outcomes of dasatinib treatment in CML.

The main weakness of this study is the reduced sample size, which limits the statistical power. Taking into account the large number of SNPs included in the study and the different analysis models, the Bonferroni-corrected threshold for statistical significance was rather stringent (p = 0.00041). None of the associations reported here were statistically significant if this threshold is used. However, it has to be kept in mind that CML is not a common disease and is also often treated with imatinib in the first line. Approximately one-third of cases are switched to the next-generation inhibitors (dasatinib, nilotinib, or others) due to lack of response or intolerance to this treatment regimen. We would like to emphasize that the population that participated in this study were patients treated in the first line with imatinib, and in the second (or third) line with dasatinib. Therefore, the above-presented results cannot be directly translated into the population of patients treated in the first line with dasatinib, because the studied population consisted of people who showed intolerance or progression during treatment, which may be of primary origin, regardless of the treatment used.

Another factor that may be a weakness is the fact that only a group of patients of Polish origin was taken into account in the analysis. Therefore, the results cannot be used in comparison to other ethnic groups (e.g., from Asia), whose genetic variability of the tested SNPs and frequency of their occurrence may differ significantly. To confirm this, additional analysis on different populations should be done.

Taking all into account, there is still little known about the impact of inherited changes in genes involved in pharmacokinetic processes in the case of TKI treatment of CML. Our work is one of the first to describe the influence of *ABCB1*, *ABCG2*, *PXR*, and *CAR* polymorphisms on the effects of dasatinib therapy (expressed through CCyR, PFS, OS) and toxicity of the therapy (expressed by association with hematological and non-hematological complications). Further analyses are needed to confirm these initial results.

## Data availability statement

The raw data supporting the conclusions of this article will be made available by the authors, without undue reservation.

## Ethics statement

This study was reviewed and approved by Ethical Committee of Medical University of Lodz (RNN/168/13/KE; 18 June 2013) Łódź, gen. Józefa Hallera Square 1B bioetyka@umed.lodz.pl. The patients/participants provided their written informed consent to participate in this study.

## Author contributions

KJ conceived and designed the study. AM performed the lab work and prepared the database. FC performed the statistical analysis. AM analyzed the results and drafted the manuscript. FC and KJ reviewed and edited the manuscript. All other authors provided samples and data. All authors contributed to the article and approved the submitted version.

## Funding

This work was supported by the grants of Young Hematologists Club of Polish Society of Hematology and Transfusiology Medicine.

## Acknowledgments

The authors would like to express their deep gratitude to Dr. Alessandro Martino for his involvement in the project of evaluation polymorphisms in chronic myeloid leukemia. The authors would also like to extend their thanks to Angelika Stein, technician at the DKFZ Genomic Epidemiology Group, for her patience and help.

## Conflict of interest

The authors declare that the research was conducted in the absence of any commercial or financial relationships that could be construed as a potential conflict of interest.

## Publisher’s note

All claims expressed in this article are solely those of the authors and do not necessarily represent those of their affiliated organizations, or those of the publisher, the editors and the reviewers. Any product that may be evaluated in this article, or claim that may be made by its manufacturer, is not guaranteed or endorsed by the publisher.
